# A Robust Visual Grasping Method for Robots in Cluttered and Stacked Scenes

**DOI:** 10.3390/s26144524

**Published:** 2026-07-16

**Authors:** Zhiqiang Gao, Mengqi Li, Huihui Bai, Jinze Li, Sifan Li, Jing Han, Zhengkai Wang

**Affiliations:** 1 Department of Automation, Taiyuan Institute of Technology, Taiyuan 030008, China; gaozhiqiang@tit.edu.cn (Z.G.); 232036127@tit.edu.cn (M.L.); baihuihui@tit.edu.cn (H.B.); 242039123@tit.edu.cn (J.L.); 242033214@tit.edu.cn (S.L.); 2 College of Mechatronic Engineering, North University of China, Taiyuan 030051, China; 13327555469@163.com

**Keywords:** visual grasping, pose estimation, cluttered stacking scenes, SAM-FoundationPose, iterative closed-loop

## Abstract

In complex backgrounds and under severe occlusions, the accuracy of vision-based robotic grasping pose estimation decreases significantly, further making objects difficult to manipulate and grasp. This paper proposes an iterative closed-loop optimization framework that deeply couples SAM with FoundationPose. The framework breaks through the open-loop logic bottleneck of “segmentation first, then estimation” found in traditional vision algorithms and constructs a mask correction mechanism based on rendered projection. By performing 3D rendering of the initially estimated 6D pose, a geometric prior mask of the object is generated and then fed back into SAM’s prompt encoder, thereby guiding the model to achieve pixel-level refinement of the target’s boundary in the next perception cycle. Meanwhile, to overcome the blind spots of a single metric, the framework designs a multi-dimensional confidence assessment module that integrates both the 2D image domain and the 3D geometric domain to comprehensively evaluate the reliability of the current pose. The SAM prior, the closed-loop iterative mechanism, and the multi-dimensional confidence assessment module work in synergy to form a complete optimization loop. In robustness experiments on pose estimation under cluttered and stacked scenes, the proposed method achieves an overall ADD-S recall rate of 91.7%, with the average translation and rotation errors reduced to as low as 3.5 mm and 2.1°. In 200 real-world robotic grasping verification trials, the overall grasping success rate reaches 96.5%. These experimental results demonstrate the effectiveness and enhanced robustness of the proposed closed-loop optimization framework in the tested unstructured environments.

## 1. Introduction

With the deepening evolution of intelligent manufacturing and collaborative robots, the operational capability of robotic arms in unstructured environments such as workpiece sorting and precision assembly has become a research focus in perception technology [1,2]. As the foundation for robots to perform physical interactions, the accuracy of vision-based 6D pose estimation of target objects directly determines the success or failure of grasping tasks [3,4]. Existing approaches to 6D pose estimation can be broadly categorized into three classes: traditional methods based on geometric optimization, regression methods based on deep learning, and methods based on rendering and matching [5,6]. Traditional methods, such as the Iterative Closest Point(ICP) algorithm and its variants, rely on local geometric gradients for point cloud registration and are highly susceptible to local optima in the absence of texture or under severe occlusions [7]. PnP-type methods, although computationally efficient, are highly sensitive to the quality of feature point matching and lack sufficient robustness [8]. Deep learning methods represented by PoseCNN and DenseFusion directly regress pose parameters or establish pixel-level dense correspondences through end-to-end networks, achieving significant breakthroughs in accuracy [9,10,11]. In recent years, many more advanced methods have further expanded this field. For instance, methods based on coordinate or dense correspondences, such as PVNet [12] and GDR-Net [13], have demonstrated high pose estimation accuracy in structured environments by establishing local geometric mappings. To fully exploit geometric features in 3D space, FFB6D [14] employs a fusion network to combine RGB and point cloud features at the pixel level. In addition, frameworks such as CosyPose [15] and ResFuNet [16] introduce a Render-and-Compare mechanism, exhibiting strong cross-category zero-shot generalization ability by evaluating the feature similarity between rendered views and real observations. However, their generalization capability is heavily constrained by the coverage of training data. Wang et al. [17] attempted to expand the perceptual dimension through viewpoint classification networks and template matching; however, this approach relies on scene-specific pre-trained models and exhibits limited performance under varying illumination or dynamic environments. Liu et al. proposed an RGB-D keypoint cloud estimation scheme that excels in lightweight deployment and real-time performance, yet it remains fundamentally a serial open-loop control and lacks the capability for feedback correction of preceding errors [18].

All the above existing segmentation-assisted or forward inference methods share a common limitation: they fundamentally adopt a unidirectional, serial open-loop control architecture. Under such a framework, there is no effective feedback and interaction between 2D semantic information and 3D spatial geometry. Once the front-end perception stage (such as object detection or image segmentation) generates noisy and erroneous masks caused by severe object occlusion, cluttered backgrounds, or low-contrast edges, such errors will be propagated unidirectionally and amplified gradually in subsequent stages. This ultimately leads to inaccurate outputs from the downstream pose estimation engine, resulting in localization failure or pose drift. The entire pipeline completely lacks the ability of self-inspection and error correction.

The emergence of visual foundation models has offered new perspectives for overcoming perception challenges. The Segment Anything Model (SAM), with its exceptional zero-shot generalization capability, enables pixel-level extraction of target masks from complex backgrounds, providing a powerful visual front end for filtering out background interference and addressing object recognition difficulties [19]. However, SAM inherently operates within the realm of 2D semantic perception and lacks the capability for pose estimation in 3D geometric space. Meanwhile, the FoundationPose model introduced at CVPR 2024 unifies the model-based and model-free technical routes by integrating neural implicit representations with prior knowledge acquired from large-scale synthetic data training, demonstrating strong cross-object transfer potential. Nevertheless, when handling highly dynamic backgrounds or occluded scenes, its single feed-forward inference results remain susceptible to the quality of initial perception, leading to localization drift [20].

To effectively mitigate the problem of perception degradation caused by unidirectional information flow at the algorithmic level, this paper does not simply patch together visual foundation models, but constructs a cross-dimensional bidirectional closed-loop collaborative optimization mechanism from the perspective of geometric mechanism. The core logic of this mechanism is to eliminate the local ambiguity of pure 2D visual features by utilizing the spatial geometric constraints of the target rigid body. The system reprojects the imperfect 3D pose estimated in the current iteration back to the 2D pixel plane to synthesize a Guided Mask with ideal geometric topology. As a definite boundary constraint, this mask is reversely injected into the prompt encoder of the large semantic model (SAM), forcing the front-end perception module to perform feature reconstruction and pixel-level boundary refinement in regions with blurred edges and weak contrast. Through such cross-dimensional feedback and interaction, the system connects the original two isolated large models into a dynamically converging self-closed-loop optimization system. Experiments demonstrate that this method not only effectively improves pose estimation accuracy in complex scenes but also provides more robust algorithmic support for autonomous robotic grasping.

## 2. Methods

In complex and heavily occluded scenes, traditional unidirectional feed-forward 6D pose estimation algorithms are susceptible to background interference or the absence of local features, resulting in pose prediction deviations. To address this issue, this paper proposes an iterative closed-loop fusion pose estimation framework based on visual foundation models, namely SAM and FoundationPose. This section first briefly introduces the principles of the adopted foundation models, followed by a detailed exposition of the proposed core system architecture and the design of each module.

### 2.1. Principles and Applications of SAM

**Figure 1:**
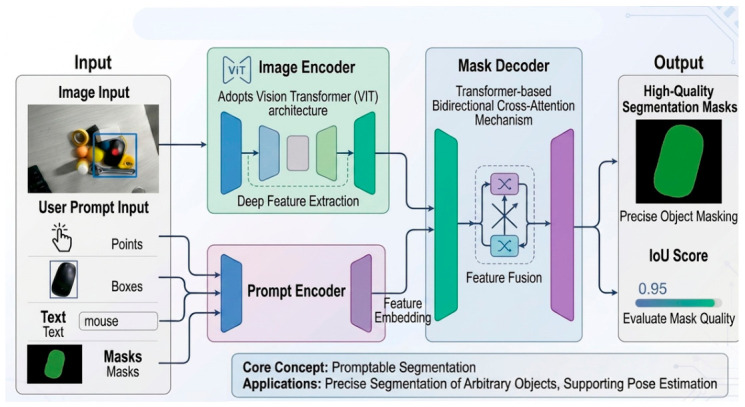
Workflow diagram of SAM.

SAM is a computer vision foundation model developed by Meta AI Research. Its core advantage lies in its powerful zero-shot generalization capability, which enables high-quality zero-shot segmentation of arbitrary objects in unseen scenes through prompts such as points, boxes, and text. SAM adopts a three-component collaborative architecture consisting of an image encoder, a prompt encoder, and a mask decoder, which work in concert to complete the entire process from image input to segmentation mask output, as illustrated in Figure 1. Specifically, the image encoder employs a Vision Transformer (ViT) architecture [21] to extract deep image features; the prompt encoder converts user-provided prompts into feature embeddings. And the mask decoder, based on the bidirectional cross-attention mechanism of Transformers [22], not only fuses the above features to generate high-quality object masks, but also produces an IoU (Intersection over Union) score for each mask to evaluate mask quality.

In this study, SAM primarily serves as a high-precision mask generator at the front end. For an input RGB image with a complex background $I\in R^{H\times W\times 3}$, SAM incorporates specific prompt information P (such as the guided mask fed back from the previous iteration) and outputs the foreground mask matrix M of the target object:

(1) $$M=f_{SAM}(I,P)$$

Compared with traditional segmentation models that struggle to accurately extract object contours in complex scenes, SAM demonstrates strong robustness in handling challenging scenarios such as stacked objects and low-contrast regions. It offers high practical value in real-world applications including autonomous driving and complex robotic environment perception, and also provides a solid technical foundation for the pose estimation research presented in this paper.

### 2.2. Principles and Applications of FoundationPose

FoundationPose is a unified high-precision 6D pose estimation and tracking foundation model that requires no training on specific objects and supports zero-shot generalization. Taking an RGB-D image and the 3D model of the target object as input, the model fuses the rendering and comparison mechanism of neural implicit representations [23,24] to output the 6D pose transformation matrix T of the object in the camera coordinate system.

The pose matrix T belongs to the three-dimensional special Euclidean group SE(3) and consists of a rotation matrix R∈SO(3) and a translation vector $t\in R^{3}$:

(2) $$T=\left[\begin{array}{cc} R & t\\ 0 & 1 \end{array}\right]\in SE(3)$$

Compared with traditional pose estimation methods such as PnP, ICP, and PoseCNN, FoundationPose demonstrates high robustness and zero-shot generalization capability in standard scenes, providing a powerful pose inference foundation for autonomous robotic grasping [25]. However, FoundationPose essentially remains a single feed-forward architecture, where its pose output is heavily dependent on the quality of the input observations. When confronted with severe occlusions or complex background interference, local appearance ambiguities caused by sensor noise or occlusions can directly affect the final inference results, and the error lacks any built-in self-inspection and correction mechanism. This non-negligible open-loop limitation constitutes the core motivation of this study [26]. To address this, we introduce a closed-loop feedback mechanism that renders the inferred pose results back into geometric priors and re-injects them into the front-end perception module, thereby endowing the system with self-inspection and correction capabilities and breaking through the open-loop constraints.

### 2.3. SAM-FoundationPose Closed-Loop Fusion Framework

Traditional deep learning-based 6D pose estimation methods [27,28,29] mainly adopt a serial, unidirectional open-loop paradigm of “segmentation first, estimation second”. Under this architecture, the main problem lies in the information flow disruption and asymmetry between 2D semantic representations and 3D spatial geometry. When dealing with extreme visual noise, severe physical occlusions, or low-contrast cluttered boundaries, the front-end 2D segmentation network often outputs “high-entropy masks” containing numerous non-target pixels, while the downstream 3D pose inference engine heavily relies on the low entropy and purity of the input feature domain. Due to the lack of an inherent corrective feedback path, this mismatch between spatial and semantic features propagates irreversibly and amplifies exponentially along the perception chain, ultimately causing the pose hypothesis to fall into local minima in space or suffer catastrophic localization failure.

To address the perception degradation bottleneck caused by information asymmetry at the underlying algorithmic level, this paper does not pursue a simple integration of visual models, but constructs a cross-dimensional bidirectional closed-loop collaborative optimization mechanism from a geometric perspective. The core algorithmic logic of this mechanism is to utilize the spatial geometric invariance constraints of rigid target objects to resolve the local ambiguity inherent in pure 2D visual features. Specifically, the system reprojects the imperfect 3D pose estimated in the current iteration back to the 2D pixel topological domain, thereby generating a guided mask with ideal geometric topology. This mask essentially acts as a deterministic nonlinear boundary constraint formed by dimension-reduced mapping from the 3D physical spatial domain to the 2D image domain. By feeding it back into the prompt encoder of the large vision foundation model, the front-end perception module is forced to perform feature reconstruction and pixel-level boundary refinement in regions with edge conflicts and low contrast. Thus, through such cross-dimensional information feedback and coupling, the system cascades the original two isolated modules into a dynamically converging adaptive optimization system.

#### 2.3.1. Overall Framework Design

**Figure 2:**
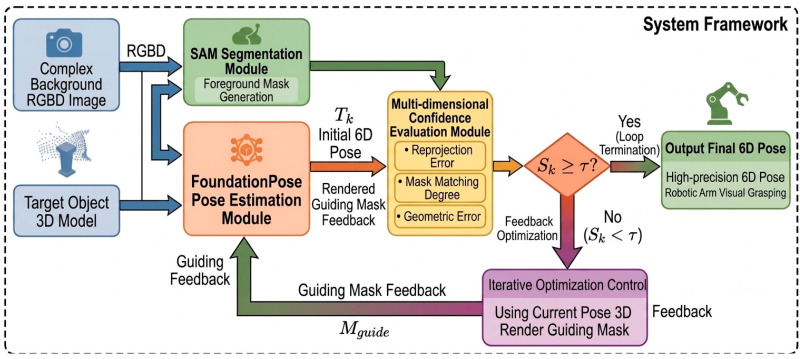
Iterative closed-loop fusion framework based on SAM and FoundationPose.

The mathematical principle and overall algorithm layout of the closed-loop optimization framework proposed in this study are illustrated in Figure 2. The global optimization objective can be regarded as a joint estimation problem over the visual mask space M and the Special Euclidean group SE(3) for rigid-body pose representation. Given an input RGB-D observation pair (I,D) and an existing geometric model M_3D_ of the target object, the system initialization layer activates the front-end segmentation encoder using a heuristic prompt bounding box to generate an initial foreground mask $M_{0}\in M$. This compact semantic mask structurally constrains the optimization search space of the pose inference engine, yielding a zero-th order pose hypothesis $T_{0}\in SE\left(3\right)$. To systematically manage the iterative process and ensure the convergence stability of the system, we introduce a multi-dimensional confidence evaluation module immediately after the pose inference layer. Serving as a cross-domain verification engine, this module comprehensively evaluates the physical and visual consistency of the current pose hypothesis $T_{k}$ by simultaneously calculating the 2D pixel reprojection error, global topological mask similarity, and 3D surface geometric deviation. The unified confidence score $S_{k}$ acts as a strict algorithmic gate to regulate the pipeline flow:

(1) If $S_{k}\geq \tau$ (where τ denotes the preset high-precision convergence threshold), the currently estimated pose is regarded as a highly optimized and reliable solution within the search space. The system terminates the closed-loop optimization and directly outputs the final pose $T^{*}=T_{k}$, which is used to drive the trajectory planning of the robot arm end-effector and perform actual physical visual grasping.
(2) If $S_{k}<\tau$, it indicates that the current pose hypothesis has fallen into a local minimum due to severe clutter or occlusion. The system immediately triggers the iterative optimization control stage. The imperfect 3D pose $T_{k}$ is fed into a forward projection operator Φ to synthesize a refined geometric guided mask $M_{guide}$:
  (3) $$P_{3D}=R_{k}X_{3D}+t_{k}$$
  (4) $$M_{guide}=\psi \left(\pi \left(KP_{3D}\right)\right)$$
  where $R_{k}\in SO\left(3\right)$ and $t_{k}\in R^{3}$ denote the rotation matrix and translation vector of the current rigid-body pose T_k_, respectively. $X_{3D}\in R^{3\times N}$ represents the vertex coordinates of the 3D object model, $P_{3D}$ denotes the 3D point cloud transformed into the camera coordinate system, and K is the camera intrinsic matrix. $\pi \left(\cdot \right)$ denotes the perspective dehomogenization projection function, while $\psi \left(\cdot \right)$ is the rasterization operator that converts the projected pixel set into a binary topological mask.

#### 2.3.2. Dynamic Prompt-Based SAM Segmentation Module

In this closed-loop framework, the SAM segmentation module serves as a front-end high-fidelity region extractor. Unlike traditional static semantic segmentation networks, this module fully exploits the promptable interactive nature of the SAM architecture and incorporates two distinct working mechanisms—initialization with heuristic prompts and guidance via iteratively rendered masks—to address the challenge of severe occlusions in complex scenes.

When the system first encounters a complex RGB-D image *I*, to activate SAM’s zero-shot segmentation capability, this module introduces heuristic prompts $P_{init}$ by employing the lightweight 2D object detection algorithm YOLOv5n to generate a coarse 2D bounding box containing the target object as the prompt input [30].Considering that a pure 2D object detector may suffer from missed detections under severe occlusions or extreme background confusion, which would result in the loss of initial prompts, this module incorporates a depth-prior-based fallback mechanism to ensure the continuity of the front-end perception pipeline. When YOLOv5n fails to produce a valid bounding box, the system automatically switches to a depth-guided mode: it performs fast Euclidean clustering on the cluttered point cloud within the workspace using the input depth map, extracts the 3D centroids of potential protruding objects, and reprojects them onto the 2D pixel plane to serve as point prompts for SAM. This dual heuristic strategy that combines RGB-D heterogeneous information effectively compensates for the robustness shortcomings of a single 2D detection front end under extreme conditions, thereby ensuring that the closed-loop iterative framework can be reliably triggered.

Let $\varepsilon_{img}$ denote the image encoder of SAM, $\varepsilon_{prompt}$ the prompt encoder, and $D_{mask}$ the mask decoder. The generation process of the initial mask $M_{0}$ can be mathematically formalized as:

(5) $$F_{I}=\varepsilon_{\mathrm{img}}(I)$$

(6) $$F_{P_{0}}=\varepsilon_{prompt}(P_{init})$$

(7) $$M_{0}=D_{mask}(F_{I},F_{P_{0}})$$

The mask generated at this stage can effectively remove the majority of background point clouds and irrelevant textures, thereby tightly constraining the search space of the subsequent FoundationPose estimation module around the target object. In practical grasping scenarios, however, the initial mask generated solely by a single-shot heuristic prompt often falls short of ideal segmentation quality. When the target object is severely occluded or its color closely resembles the background, SAM’s segmentation results may suffer from over-segmentation or under-segmentation, and the single feed-forward mechanism will irreversibly propagate such errors to the pose estimation module, leading to the accumulation of pose errors [31,32,33].

To overcome this limitation, this paper deeply embeds this module into the closed-loop feedback pipeline. When the system enters the *k*-th iteration (k ≥ 1), this module receives the guided mask from the iterative optimization control module $M_{guided}^{\left(k-1\right)}$. This guided mask is rendered from the imperfect pose $T_{k-1}$ estimated in the (k − 1)-th iteration and the 3D model, and it provides the ideal geometric topology of the target under the current viewpoint. Injecting $M_{guided}^{\left(k-1\right)}$ as a spatial prior prompt into the system can force SAM to re-correct the segmentation boundaries in edge-ambiguous regions. The mask generation equation for the *k*-th iteration is updated as:

(8) $$F_{P_{k}}=\varepsilon_{prompt}(P_{init},M_{guided}^{\left(k-1\right)})$$

(9) $$M_{k}=D_{mask}(F_{1},F_{P_{k}})$$

By implementing the closed-loop interaction mechanism described above, the SAM segmentation module evolves from an isolated preprocessing step into a dynamically self-optimizing perception module. After the refined mask $M_{k}$ it produces is fed into the FoundationPose pose estimation module, it can substantially reduce the interference of outliers and enhance the robustness of feature matching, thereby laying a foundation for achieving high-precision robotic grasping.

#### 2.3.3. FoundationPose Pose Estimation Module

After generating the high-precision foreground mask of the target object, the system employs FoundationPose as the core 6D pose inference engine. Traditional single networks often require lengthy fine-tuning for specific objects, whereas FoundationPose, as a visual foundation model, leverages its powerful large-scale generalization prior to directly perform zero-shot pose estimation for novel objects. Within this closed-loop framework, the module first extracts local features through mask-guided region-of-interest cropping, and then generates and selects the optimal 6D pose hypotheses via the rendering and comparison mechanism [34,35].

In the *k*-th iteration, this module receives the global observation data from the environment (i.e., the RGB image *I* and the depth map *D*), the 3D model $M_{3D}$ of the target object, and the current frame foreground mask $M_{k}$ output by the SAM module. To eliminate the interference of complex backgrounds on pose estimation, the module first performs a bitwise AND operation between $M_{k}$ and the global observation data to extract local observation features $I_{ROI}$ and $D_{ROI}$ that are strictly constrained within the target region:

(10) $$I_{ROI}=I⊙M_{k}$$

(11) $$D_{ROI}=D⊙M_{k}$$

Through this mask-guided feature space constraint, the feature extraction of the FoundationPose module is highly concentrated on the effective pixels of the target object. This significantly reduces the probability of false feature matches caused by external occluding objects or similar background textures, thereby providing a relatively clean input source for subsequent pose estimation.

FoundationPose internally adopts a network architecture that combines neural implicit representations with rendering-based comparison. Let $F_{pose}$ denote the core inference network of FoundationPose. By performing deep feature extraction on the input local observation features $\left(I_{ROI},D_{ROI}\right)$ and the 3D model $M_{3D}$, the network generates a set of initial 6D pose hypotheses within the vast pose search space. Through the rendering and comparison mechanism, the feature similarity between the rendered views of the 3D model under each hypothesized pose and the actual 2D observations is evaluated at the feature level, and the highest-scoring pose is output as the estimated result $T_{k}$ for the current round. This process can be mathematically abstracted as:

(12) $$T_{k}=F_{pose}(I_{ROI},D_{ROI},M_{3D})$$

where $T_{k}$ represents the 6D rigid-body transformation matrix of the target object in the camera coordinate system, belonging to the three-dimensional special Euclidean group SE(3). It consists of a 3 × 3 orthogonal matrix $R_{k}\in SO(3)$ representing rotation and a 3 × 1 vector $t_{k}\in R^{3}$ representing translation:

(13) $$T_{k}=\left[\begin{array}{cc} R_{k} & t_{k}\\ 0 & 1 \end{array}\right]$$

In traditional open-loop applications, the output of FoundationPose is taken directly as the final result. However, this output is constrained by the inherent limitation of single feed-forward inference: when the initial mask *M*_0_ contains noise, the *F_pose_* output by *T*_0_ is highly prone to falling into a local optimum. Within the closed-loop framework constructed in this study, as the number of iterations k increases, the fed-back mask *M_k_* increasingly approaches the true physical contours in terms of boundary delineation. The FoundationPose module performs re-estimation using the progressively purified ***I_ROI_*** and *D_ROI_*, enabling the output pose *T_k_* to gradually converge toward a highly accurate estimation in both translation and rotation dimensions. The computed current pose *T_k_* is then fed into the multi-dimensional confidence assessment module to determine whether to initiate the next round of mask refinement and pose iteration.

#### 2.3.4. Multi-Dimensional Confidence Assessment Module

In the transition from a feed-forward network to a closed-loop iterative system, accurately evaluating the reliability of the current pose and deciding whether to terminate the iteration lies at the core of the entire framework. Traditional single evaluation metrics often suffer from inherent limitations. For example, relying solely on 2D re-projection error can easily fall into local optima in textureless regions, while relying solely on 3D depth error struggles to handle the pose ambiguity of symmetric objects. To overcome these limitations, this framework designs a multi-dimensional confidence assessment module that integrates both the 2D image domain and the 3D geometric domain. The module comprises three mutually orthogonal and complementary sub-metrics: re-projection error, mask matching degree, and geometric error [36].

The re-projection error is used to measure the alignment accuracy of the pose on the 2D pixel plane, primarily constraining the translation of the target object along the X- and Y-axes as well as the pitch or yaw angles. Let the set of sampled points on the surface of the target object’s 3D model be denoted as $P_{3D}={\left\{p_{i}\right\}}_{i=1}^{N}\in R^{3}$. In the *k*-th iteration, given the known camera intrinsic matrix K, the system projects the 3D point cloud onto the 2D pixel plane according to the current predicted pose $T_{k}=[R_{k}|t_{k}]$. Let π(⋅) be the perspective projection function from homogeneous coordinates to pixel coordinates. Then the average re-projection error $u_{i}^{proj}$ between the projected point $u_{i}^{obs}$ and the matched actual observed feature point $E_{reproj}^{\left(k\right)}$ in the image is defined as:

(14) $$E_{reproj}^{\left(k\right)}=\frac{1}{N}\overset{N}{\underset{i=1}{\Sigma }}\left\|\pi (K(R_{k}p_{i}+t_{k}))\right.-{\left.u_{i}^{obs}\right\|}_{2}$$

where ||∙||_2_ denotes the L2 norm. A smaller $E_{reproj}^{\left(k\right)}$ indicates a higher degree of alignment of the 2D visual features.

To constrain the pose from a global topological perspective, this module introduces the mask matching degree. It primarily evaluates the plausibility of the predicted pose in terms of the object’s overall contour and boundary scale. Using the current predicted pose $T_{k}$ and the 3D model, the system renders a predicted foreground mask $M_{render}^{\left(k\right)}$ of the target object from the camera viewpoint. The Intersection over Union (IoU) between this rendered mask and the observed mask $M_{k}$ output by the current round of the SAM module is then computed as:

(15) $$S_{iou}^{\left(k\right)}=\frac{\left|M_{render}^{\left(k\right)}\cap M_{k}\right|}{\left|M_{render}^{\left(k\right)}\cup M_{k}\right|}$$

The value of $S_{iou}^{\left(k\right)}$ ranges from 0 to 1. A higher score indicates a greater consistency between the object contour under the predicted pose and the actual observed contour, effectively preventing the algorithm from producing scale divergence under severe occlusions.

Although the above two 2D-domain metrics can effectively constrain the planar pose, they lack sensitivity to depth translation along the camera’s optical axis (*Z*-axis). Therefore, the module introduces a depth-map-based geometric error to perform verification directly in the 3D physical space. Let $D_{render}^{\left(k\right)}$ be the depth map rendered using $T_{k}$, and $D_{obs}$ be the observed depth map actually captured by the depth camera. To exclude background interference, the error computation is performed only within the valid overlap region $\Omega =M_{render}^{\left(k\right)}\cap M_{k}$. The geometric error $E_{geo}^{\left(k\right)}$ is quantified using the root mean square error:

(16) $$E_{geo}^{\left(k\right)}=\sqrt{\frac{1}{\left|\Omega \right|}\sum_{x\in \Omega }{\left(D_{render}^{\left(k\right)}(x)-D_{obs}(x)\right)}^{2}}$$

where *x* represents the pixel coordinates, and |*Ω*| denotes the total number of pixels within the valid region. The geometric error ensures a tight alignment between the predicted pose and the true 3D surface of the object.

To establish a unified convergence criterion, the system fuses the above three metrics, which have different dimensions, into a single scalar score. Since the re-projection error and the geometric error are the smaller the better, while the mask matching degree is the larger the better, the system first applies a bounded normalization to the two error metrics using a nonlinear negative exponential function N(x)=exp(−λx) (where λ is a tuning coefficient). The final comprehensive confidence score $S_{k}$ for the k-th iteration is computed as follows:

(17) $$S_{k}=\omega_{1}\exp(-\lambda_{1}E_{reproj}^{\left(k\right)})+\omega_{2}S_{iou}^{\left(k\right)}+\omega_{3}\exp(-\lambda_{2}E_{geo}^{\left(k\right)})$$

where ω_1_, ω_2_, and ω_3_ are the weight coefficients of the respective metrics, satisfying $\omega_{1}+\omega_{2}+\omega_{3}=1$. In the specific implementation of this study, the negative exponential normalization adjustment coefficient is set to λ = 1.0. The weight coefficients of each sub-index are configured as follows: the weight of reprojection error ω_1_ = 0.3, the weight of mask matching degree ω_2_ = 0.4, and the weight of geometric depth error ω_3_ = 0.3. The above combination of parameters and weights is optimized through multiple groups of grid search experiments on a subset of the self-built dataset (validation set). During the tuning process, it is found that setting ω_2_ to a relatively high weight (0.4) can significantly improve the system’s ability to capture boundaries under physical occlusion and contour changes of target object edges. Meanwhile, cooperating with ω_1_ and ω_3_ can effectively ensure the convergence accuracy of the system in all translation and rotation dimensions of the 3D physical space. The comprehensive confidence score $S_{k}\in \left[0,1\right]$ can comprehensively and objectively reflect the accuracy of the current 6D pose. This score is then passed to the iterative optimization control module, serving as the sole trigger condition for the system’s closed-loop operation.

#### 2.3.5. Iterative Optimization Control

The iterative optimization control module serves as the core hub for controlling the closed-loop feedback pipeline of the system. In the feed-forward networks of traditional visual pose estimation, errors from front-end perception and segmentation are irreversibly propagated downstream and amplified. To break through this unidirectional data flow barrier, this module acts as a logic gate that governs the convergence state of the system based on the comprehensive confidence score output by the multi-dimensional confidence assessment module. When the comprehensive confidence score falls below the preset qualification threshold, it indicates that the currently inferred pose suffers from local optimum collapse caused by extreme occlusions or severe background confusion, and the system immediately activates the closed-loop feedback optimization loop. Once the iterative optimization stage is triggered, the module first extracts the imperfect 6D pose matrix output by FoundationPose in the current round and deeply couples it with the 3D model of the target object. Using a graphics rendering engine, the system reprojects the current 3D pose hypothesis back onto the 2D camera observation plane, thereby generating a guided mask that possesses the ideal geometric topology of the target. The essence of this rendering feedback operation is the dimensionality reduction in error correction information from the 3D spatial domain into a 2D geometric prior. Subsequently, this guided mask is injected in reverse into the prompt encoder of the front-end SAM segmentation module as a dynamic spatial prompt.

Under this cross-dimensional rendering guidance, SAM is forced to re-constrain its feature matching scope in edge-ambiguous and highly interfered regions, thereby effectively eliminating the interference of erroneous background textures and outputting a pixel-level refined high-fidelity foreground mask. Subsequently, the FoundationPose module receives the purified effective region features and performs a new round of pose estimation, driving the output pose toward an accurate alignment in both translation and rotation dimensions. The system undergoes successive linear iterations within the bidirectional closed-loop pipeline based on rendering feedback until the multi-dimensional comprehensive confidence score of the output pose meets the threshold criterion. At this point, the iterative control mechanism terminates and outputs the final high-precision 6D pose.

The detailed execution procedure of the entire SAM-FoundationPose iterative optimization control is presented in Table 1.

## 3. Experiments and Analysis

To thoroughly validate the accuracy and robustness of the 6D pose estimation method under the SAM-FoundationPose iterative closed-loop fusion framework, this section takes ten common objects—including a computer mouse, tape measure, adhesive tape, and earphones—as experimental targets. Using the constructed robotic arm visual grasping platform, a series of experiments are conducted successively: object pose estimation in cluttered but non-stacked scenes, object pose estimation in cluttered and stacked scenes, comparative experiments with different estimation methods, core module ablation experiments, and robotic arm visual grasping verification experiments in real-world scenarios. Through the above series of experiments, the self-correction capability of the proposed closed-loop framework when facing extreme visual interference and severe occlusions is comprehensively validated, effectively demonstrating the decisive role of introducing the iterative rendered feedback mechanism in improving 6D pose estimation accuracy and ensuring reliable robotic grasping in unstructured environments.

### 3.1. Platform Setup and Experimental Preparation

**Figure 3:**
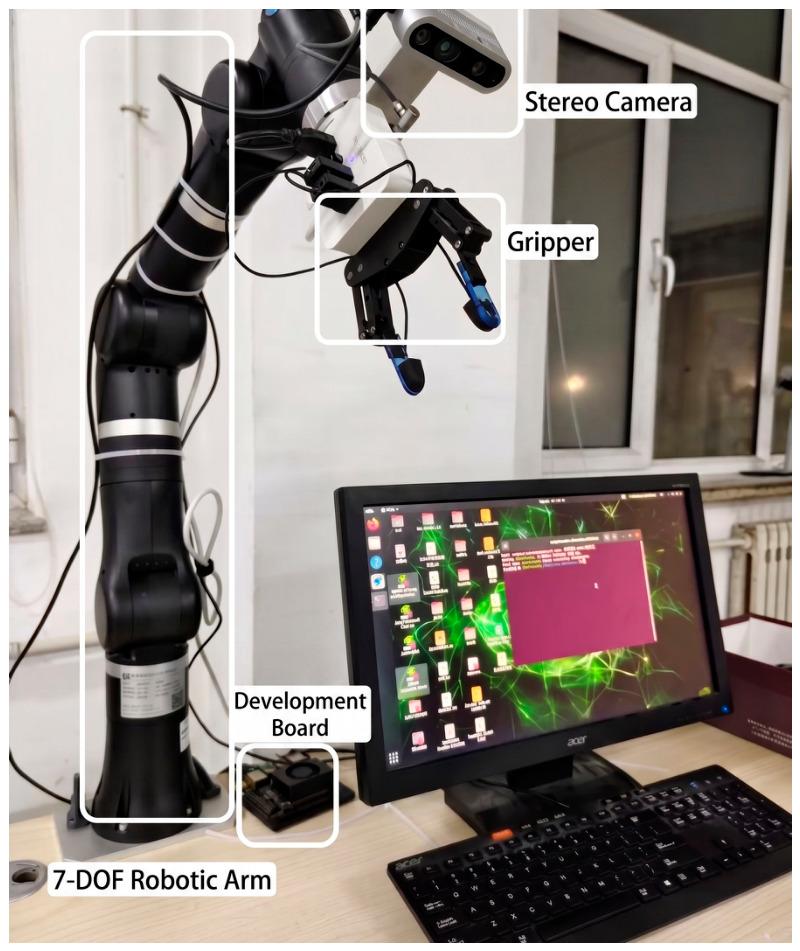
Experimental hardware system composition.

The experimental hardware system consists of three main components: a vision sensor, an edge computing core, and a collaborative robotic arm. As shown in Figure 3, an Intel RealSense D435i depth camera (Intel Corporation, Santa Clara, CA, USA) is selected as the vision sensor, providing color features for the SAM segmentation module and supplying 3D spatial geometric information for the FoundationPose and multi-dimensional confidence assessment modules. An NVIDIA Jetson NX (NVIDIA, Santa Clara, CA, USA) is adopted as the computing core to handle visual inference tasks, and a GEN72 series ultra-lightweight collaborative robotic arm (Realman Intelligent Technology Co., Ltd., Beijing, China) serves as the grasping verification platform to execute physical manipulation tasks after pose output.

**Figure 4:**
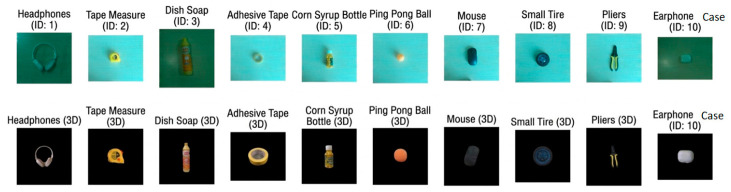
Comparison of real images (**top**) and 3D models (**bottom**) of the ten objects.

In terms of experimental targets, ten representative everyday objects were selected, including a computer mouse, tape measure, adhesive tape, earphones, a ping-pong ball, etc. The visual images and 3D geometric models of these objects are shown in Figure 4. The primary rationale for selecting these objects lies in their strong representativeness in terms of geometric shape complexity (e.g., cylinders, flat objects, irregular geometries), surface texture characteristics (e.g., smooth and reflective, richly textured), and size range (from a small ping-pong ball to a large detergent bottle), enabling effective evaluation of the accuracy and robustness of the proposed framework for object pose estimation in complex scenes.

It is particularly emphasized that the closed-loop optimization framework of SAM-FoundationPose proposed in this paper is a completely training-free zero-shot perception method. Fundamentally different from traditional deep learning methods (such as PoseCNN) that rely heavily on large-scale annotated real images and are prone to overfitting to specific objects, the entire pipeline of this framework only requires the standard 3D model of the target rigid body as the sole geometric prior. Therefore, the 10 categories of rigid objects selected in this experiment are not used for fitting and training, but to evaluate the generalization performance across different geometric topologies. These 10 types of objects precisely cover objects with distinct characteristics, including texture-less symmetric objects (table tennis balls), highly reflective specular objects (earphone cases), irregular slender structures (pliers), and objects with rich high-frequency textures (small tires). For symmetric objects included in the experiments (such as detergent bottles, table tennis balls, etc.), the framework employs a multi-dimensional confidence evaluation module to constrain their pose uncertainty. When the pose of a symmetric object rotates around its symmetry axis, although the 3D surface geometric depth error cannot effectively detect such rotational deviation, the 2D reprojection error and the intersection over union (IoU) of the rendered mask are highly sensitive to changes in the boundary contour on the 2D plane. Through cross-validation of 2D and 3D multi-dimensional metrics, the system can accurately constrain the pose of symmetric objects across all degrees of freedom.

In terms of the underlying runtime environment, the edge computing core is equipped with the JetPack 5.1.2 operating system and configured with CUDA 11.4 and cuDNN computational acceleration libraries, fully harnessing the GPU computing power of the Jetson platform. The system’s algorithm development is primarily based on Python 3.8, and both the model’s forward inference and the closed-loop iterative optimization computations are natively deployed within the PyTorch 1.13.0 deep learning framework. To balance perception accuracy and operational efficiency under constrained edge computing resources, the system adopts the lightweight segmentation foundation model MobileSAM at the front end and utilizes PyTorch’s native automatic mixed precision capability to accelerate the tensor operations of FoundationPose.

It should be noted that since the proposed framework adopts a training-free, zero-shot perception paradigm, no training data collection is required in the experiments, and there is no division between training and test sets. Regarding the specific data scale and number of experiments: In the non-stacked cluttered scene tests, 10 categories of target objects were used. By randomly varying the placement position, orientation, and desktop background interference, a total of 100 independent test images were collected and evaluated (10 images per object category), with a physical occlusion rate of 0%. In the dense cluttered stacking scene tests, the target objects were severely occluded, with the surface occlusion ratio typically ranging from 30% to 60%. In this scenario, 20 independent and randomized repeated pose estimation tests were performed on challenging targets to analyze the dispersion and probability density distribution of the errors. In the final real robotic arm grasping verification experiments, a total of 200 physical grasping attempts were conducted (10 object categories, with 20 independent repeated tests per category). Before each test, the stacking layout of the objects in the bin was thoroughly reshuffled to ensure sample randomness.

To ensure the absolute objectivity of the evaluation metrics, a standard pipeline for acquiring 6D pose ground truth is established in this experiment. In each test under cluttered and stacked scenes, an initial coarse pose is first estimated using calibration markers attached to the bottom of the target object combined with high-precision camera intrinsic parameters, under an unoccluded condition. Subsequently, dense point clouds collected from multiple views are uniformly transformed into the base coordinate system by leveraging high-precision forward kinematics data of the robotic arm and the hand-eye calibration matrix. Finally, fine registration between the standard 3D model of the object and the scene point cloud is performed via ICP (Iterative Closest Point).

### 3.2. Object Pose Estimation in Cluttered but Non-Stacked Scenes

In practical robotic grasping applications, target objects are often subject to interference from surrounding clutter and confusion caused by similar background textures. Such environments can severely disrupt the feature extraction network’s ability to correctly identify object boundaries and surface characteristics. To validate the adaptability of the proposed algorithm in non-ideal environments, this section conducts a robustness evaluation on object pose recognition under complex background interference. The experiment aims to verify the perception accuracy of the SAM-FoundationPose framework under conditions with no external physical occlusion but with severe background color and high-frequency texture confusion. The experimental scene is set up as a desktop with complex patterns and reflective interference. The ten selected categories of target objects are randomly placed within the camera’s field of view. The core evaluation metrics employed are the ADD(-S)average recall rate (with a threshold of 2 cm), the average translation error, and the average rotation error.

#### 3.2.1. Robustness Analysis

Under visual inputs with strong background texture interference, traditional feed-forward segmentation algorithms based on edges or color gradients are highly prone to misidentifying background patterns as object contours, thereby causing the collapse of subsequent pose estimation. In the proposed framework, benefiting from the exceptional zero-shot semantic generalization capability of the SAM front end, the system can still effectively resist 2D background noise and achieve accurate separation of foreground regions.

**Figure 5:**
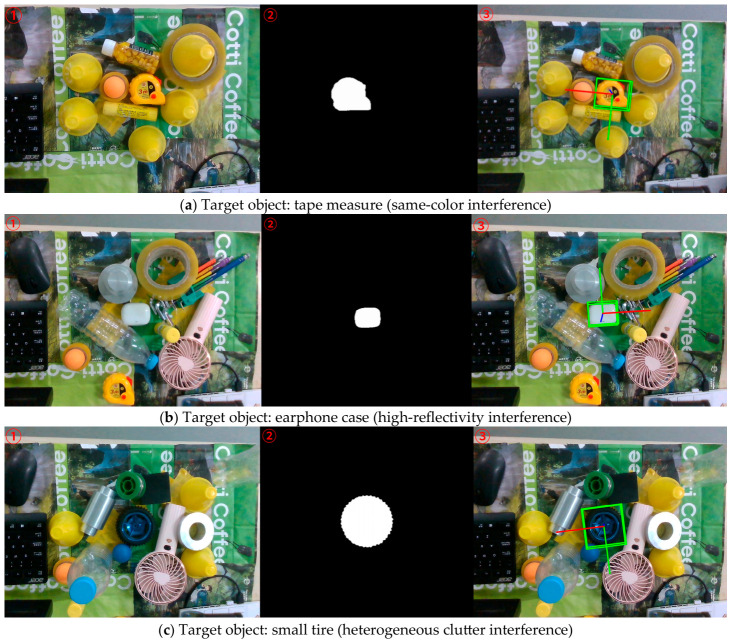
Recognition results of 6D pose estimation in three typical challenging scenarios. The 3D coordinates of the object in the camera coordinate system are denoted by x, y, and z. The red axis represents the x-axis, green represents the y-axis, and blue represents the z-axis.

To intuitively demonstrate the system’s perceptual robustness under 2D visual interference, Figure 5 presents the intermediate processing and pose rendering results for individual target objects in three typical challenging scenarios. The three scenarios are as follows. Figure 5a shows the same-color and background texture confusion scenario, with the tape measure as the target, the test environment contains background color blocks highly similar to the target color, with multiple same-color distractors scattered around. Figure 5b shows the high-reflectivity and transparent material interference scenario: focusing on the earphone case with a smooth surface, under the combined refraction of multiple transparent and reflective objects in the surroundings, the specular reflections and background color transmission on the target surface pose severe challenges to mask boundary extraction. Figure 5c shows the strong feature texture and clutter interference scenario: centering on a small tire with strong jagged textures, this scenario verifies the system’s ability to eliminate high-frequency background noise and accurately capture the effective geometric contours of the target when surrounded by scattered objects of diverse shapes.

In Figure 5, ① denotes the RGB input image from the camera, ② denotes the binary mask after segmentation by the SAM module, and ③ denotes the final rendered 6D pose output. It can be observed from Figure 5 that the system still delivers relatively high-quality perception results in the three typical challenging scenarios described above. In scenario (a), despite the color overlap between the tape measure body and the background pattern as well as interference from multiple same-color objects, the final mask generated by the system still accurately segments the target contour without any background adhesion, and the pose rendering box tightly fits the target center. In scenario (b), faced with high-reflectivity interference from the earphone case surface and surrounding objects, traditional algorithms are prone to breakage in highly reflective and bright regions, whereas the mask output by the proposed framework maintains high integrity and smoothness, demonstrating that this method effectively overcomes the 2D feature ambiguity caused by background color transmission. In scenario (c), targeting the small tire with strong jagged edges, under dense occlusion by surrounding clutter, the system still successfully locks onto the target; its mask contour faithfully restores the geometric jagged features of the tire edges, and the pose rendering box output by the system tightly fits the physical object, which verifies that when confronting high-frequency texture interference, the framework can effectively guide the model to traverse local minima through rendering feedback, ensuring convergence in the three-dimensional spatial domain.

#### 3.2.2. Positioning Accuracy Analysis

To objectively quantify the system’s anti-interference capability under complex backgrounds, the experiment collected statistics on the multi-dimensional pose errors of the ten categories of test objects in a desktop environment with strong interference. The quantitative evaluation results are presented in Table 2.

According to the quantitative statistical results in Table 2, the SAM-FoundationPose closed-loop framework demonstrates high perceptual accuracy and 3D spatial alignment capability under complex backgrounds. In comprehensive tests on ten target objects with different physical characteristics, the overall average ADD(-S) recall rate of the system reaches 97.1%, while the average translation error is controlled at a low level of 3.63 mm and the average rotation error is as low as 2.21°. When encountering samples with optical and color ambiguities, the proposed algorithm still maintains strong robustness. For instance, for the adhesive tape that is transparent and has extremely low edge contrast, the earphone case subject to specular reflections, and the tape measure deeply entangled in same-color background confusion, their translation errors are all effectively constrained to within 5.2 mm, and the recall rates remain consistently above 94%.

This result fully demonstrates that the self-optimizing closed-loop logic based on rendering feedback constructed in this paper can effectively resist 2D visual deceptions (such as high-frequency textures, similar colors, and multi-source reflections) under unstructured working conditions. Through continuous correction via rendering priors, the system achieves effective convergence in 3D physical space pose estimation, thereby establishing a reliable perception foundation for the subsequent more challenging grasping tasks involving severe occlusions and cluttered stacking scenarios.

### 3.3. Object Pose Estimation in Cluttered and Stacked Scenes

Having established the baseline performance, this section focuses on evaluating the robustness of pose estimation under extreme conditions such as severe occlusions, cluttered stacking, and complex background interference. These experiments correspond to the highly challenging robotic grasping environments encountered in real-world scenarios and also validate the core value of the proposed “iterative closed-loop fusion mechanism”.

This paper aims to test the system’s self-correction and pose optimization capabilities in the face of front-end input failures caused by multiple interfering factors. To this end, a highly cluttered experimental scene was constructed: ten objects were randomly and densely stacked in an area containing complex textures (such as a patterned tabletop), with the tape measure serving as the target object. In this scene, not only are the features of the tape measure partially occluded, but its edge contours are also severely confused with those of neighboring objects or the background.

In complex stacking scenarios, the system progressively approaches the true target through multiple rounds of rendering feedback, exhibiting the dynamic evolutionary characteristics unique to closed-loop control. At the initial stage, due to severe occlusions and background interference, the initial foreground mask generated by the SAM segmentation module contains considerable noise (e.g., misclassified edges of adjacent objects). As a result of this error, the initial 6D pose output by the FoundationPose module typically exhibits significant translational deviations or orientation flipping. If a traditional single-shot open-loop network were employed, this erroneous pose would directly lead to the failure of subsequent robotic grasping.

**Figure 6:**
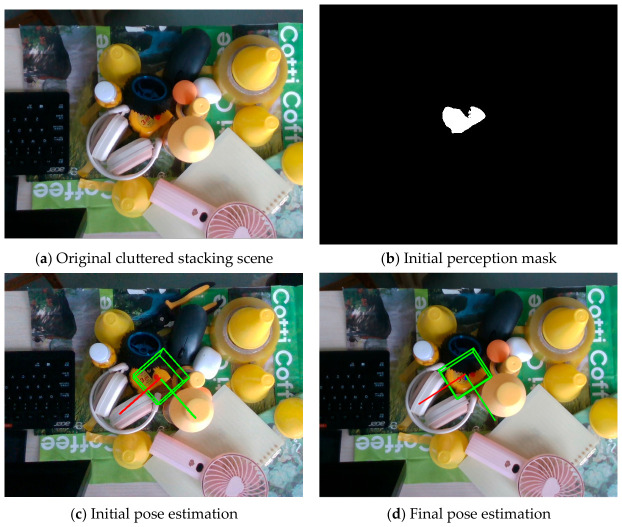
Visualization of the closed-loop correction process. The 3D coordinates of the object in the camera coordinate system are denoted by x, y, and z. The red axis represents the x-axis, green represents the y-axis, and blue represents the z-axis.

In the framework constructed in this paper, this erroneous pose is fed into the “multi-dimensional confidence assessment module.” Due to the inaccuracy of the initial pose, the mask rendered from it exhibits extremely low overlap with the actual observed mask, and the geometric re-projection error surges, causing the system’s comprehensive confidence score to fall far below the qualification threshold. At this point, the iterative optimization control mechanism is formally activated. The system uses the current erroneous pose as a prompt, renders a new target mask contour, and feeds it back to the SAM module. Under the effect of this powerful “rendering-guided mask feedback,” SAM is able to exclude background interference, refocus on the true object boundaries, and generate a refined mask with substantially improved quality. As the iteration proceeds, the prior information received by FoundationPose becomes increasingly accurate, and the output pose progressively approaches the true state until the confidence score meets the threshold and the loop is exited. The correction evolution process of the system in a real-world scenario is illustrated in Figure 6.

From Figure 6, the dynamic correction evolution process of the system can be intuitively observed. In the initial state as shown in Figure 6a, the target object, the tape measure, is occluded by surrounding clutter and exhibits strong color confusion with the yellow conical object on the left. This causes the initial perception mask output by SAM during the first forward inference as shown in Figure 6b to contain substantial noise, failing to accurately isolate the tape measure itself and displaying obvious regional adhesion with adjacent distractors. Misled by this low-quality mask, the solved initial pose as Figure 6c exhibits significant spatial translation deviation and misalignment, rendering it incapable of guiding actual physical grasping. After triggering the closed-loop correction mechanism of the framework, the system converts the deviated pose in Figure 6c into a geometric prior and guides the front-end perception in reverse. Through iterative convergence, the system ultimately succeeds in eliminating interference, and the output final pose as shown in Figure 6d achieves tight alignment with the true 3D physical boundaries of the tape measure. This intuitive visual evolution fully validates the self-correction capability of the proposed mechanism when dealing with severe occlusions and deep confusion.

**Figure 7:**
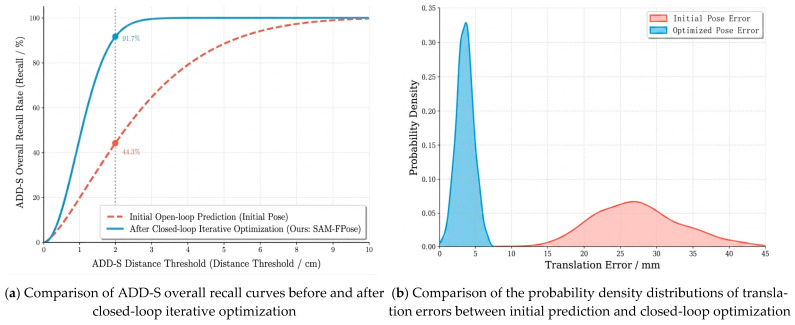
Pose error comparison and performance curves.

To verify the self-correction capability of the aforementioned closed-loop iterative mechanism when handling severely occluded samples, this section conducts a comprehensive evaluation from two dimensions: macro-level precision and recall, and micro-level error distribution analysis. The results are shown in Figure 7. To further analyze the performance in stacked scenarios, this set of experiments focuses on the individual analysis of a relatively difficult target—a tape measure—in cluttered and stacked scenes.

Figure 7a shows the comprehensive recall rate curves of the system under different ADD-S distance tolerance thresholds. In the initial open-loop prediction without feedback optimization (red curve), the system is highly prone to falling into local optima due to severe occlusions and lighting interference, achieving a recall rate of only 44.3% at the core evaluation point (2 cm threshold). In contrast, after introducing the mask rendering feedback and multi-dimensional confidence evaluation mechanism, the closed-loop iterative optimization curve (solid blue line) exhibits a steep upward trend and rapidly converges to saturation. Its recall rate at the 2 cm threshold rises to 91.7%, fully demonstrating the system’s strong capability in large-range pose rectification and adaptive correction.

To further quantitatively analyze the suppression effect of closed-loop iteration on error fluctuations from a statistical perspective, Figure 7b depicts the probability density distribution and dispersion indices of translation errors before and after the closed-loop optimization. The blue curve represents the probability density of pose errors after optimization, while the red curve represents that before optimization. It can be observed that the blue distribution features an extremely sharp peak and a narrow range, with errors almost entirely concentrated within 0–5 mm and almost no large-offset samples exceeding 10 mm. This indicates that millimeter-level accurate localization can be achieved for most objects. In contrast, the red distribution shifts significantly to the right with a low peak and a wide spread, where errors are concentrated in the range of 15–35 mm, accompanied by numerous samples with large localization deviations. This intuitively demonstrates that single-shot open-loop inference is highly susceptible to weak texture, occlusion, and specular reflection, resulting in severe pose drift.

The proposed system demonstrates high convergence efficiency in severely occluded scenes, typically reaching the confidence threshold and terminating the loop within 3 to 5 iterations (4 on average). Unlike traditional registration algorithms that rely on local geometric gradients, such as ICP, which often require dozens of iterations, the extremely fast convergence speed of the proposed framework is primarily attributed to the powerful global prior capability of the visual foundation model. In the initial 1 to 2 iterations of the closed-loop pipeline, the rendering feedback mechanism provides strong geometric constraints that contain the complete topological structure of the target, forcing SAM to instantly leap over local minima and eliminating most of the scale divergence and severe translational drift visible in Figure 7b. In the subsequent 3 to 4 iterations, the system primarily performs depth fine-tuning and pose alignment along the *Z*-axis at the sub-centimeter level, ultimately achieving the evolution from the red divergent distribution to the blue high-precision concentrated distribution shown in Figure 7b.

### 3.4. Comparative Experiments with Different Estimation Methods

To comprehensively evaluate the overall performance of the proposed SAM-FoundationPose iterative closed-loop fusion framework in complex environments, this section conducts comparative experiments between the proposed method and current mainstream and state-of-the-art 6D pose estimation algorithms on the previously constructed dataset featuring complex occlusions and stacking.

#### 3.4.1. Selection of Comparative Algorithms and Evaluation Metrics

To ensure fairness and comprehensiveness in the comparison, the following three representative baseline methods are selected for the experiment:

PoseCNN: A classic convolutional neural network-based pose estimation method using pure RGB images, often serving as a low-level baseline for pose estimation.

MegaPose: An advanced zero-shot 6D pose estimation algorithm with strong cross-category generalization capability.

Original FoundationPose: The original feed-forward network without incorporating the SAM prior mask or the closed-loop iterative mechanism proposed in this paper, serving as an ablation verification baseline.

A reasonable explanation is provided regarding the fairness and rationality of the comparison among the three aforementioned methods. First, PoseCNN represents a classic deep regression baseline, whose input is limited to pure RGB images without utilizing 3D spatial data. It is included in the comparison mainly to serve as a lower-bound baseline for classic deep regression methods lacking 3D geometric information. Second, both MegaPose and the original FoundationPose employ RGB-D data and require 3D models as geometric object priors. Our proposed method shares exactly the same input data and prior constraints with the latter two, ensuring a strictly modality-aligned comparison. Finally, to guarantee relative fairness and objectivity in the comparative study, all baseline algorithms (PoseCNN, MegaPose, and the original FoundationPose) are deployed and evaluated on the identical edge computing hardware platform (NVIDIA Jetson NX), using the unified PyTorch 1.13.0 framework and CUDA 11.4 acceleration library. None of the compared vision foundation models have been fine-tuned for any specific scene.

#### 3.4.2. Result Comparison and Analysis

The comprehensive test results of each algorithm on the ten categories of target objects under the cluttered stacking background constructed in Section 3.3 are presented in Table 3. As can be seen from the data in Table 3, the classic PoseCNN and DenseFusion suffer from a precipitous drop in performance when facing severe occlusions and cluttered backgrounds, with their ADD-S recall rates falling below 60%. This is because such methods are highly dependent on the integrity of global appearance features or point clouds; once the target contour is disrupted, the error surges dramatically. In contrast, the state-of-the-art MegaPose and the original FoundationPose demonstrate relatively strong robustness, with recall rates improved to 71.4% and 79.6%, respectively. However, as single-shot open-loop systems, they still exhibit noticeable translational deviations when confronted with highly confusing background edges.

In contrast, the SAM-FoundationPose method proposed in this paper achieves a notable advantage in accuracy metrics. Its ADD-S recall rate reaches as high as 91.7%, representing an increase of 12.1 percentage points over the original FoundationPose; the average translation and rotation errors are reduced to as low as 3.5 mm and 2.1°. This set of quantitative results fully demonstrates that, by introducing the SAM segmentation prior and closed-loop iterative optimization, the proposed method not only significantly improves the pose estimation accuracy in complex stacking scenarios but also fundamentally overcomes the robustness bottleneck of traditional open-loop methods under occlusion and interference, exhibiting clear performance superiority.

To evaluate the additional computational overhead introduced by the iterative feedback loop, we conducted a detailed runtime breakdown of the proposed SAM-FoundationPose framework on the NVIDIA Jetson NX platform. As shown in Table 3, the proposed method takes an average of 390 ms to process one object. This duration mainly consists of two stages. The first stage is initial pose estimation (approximately 210 ms), which runs once at the beginning to compute a preliminary pose. Specifically, SAM takes about 70 ms to extract the initial contour of the object, and then FoundationPose takes about 140 ms to estimate the position and orientation of the object. The second stage is iterative pose optimization (approximately 60 ms per iteration). After obtaining the initial pose, the system requires an average of 3 additional iterations to achieve the required high precision. Each subsequent iteration is much faster for two key reasons. First, image features are reused without repeated computation. Since the input image remains unchanged, the most time-consuming image feature extraction has already been completed in the first stage and can be directly reused in subsequent iterations, so updating the contour only takes about 5 ms. Second, computation is restricted to a small local region. All subsequent calculations are confined to a small area around the object; reprojection of the 3D model takes only about 5 ms, and pose refinement by FoundationPose takes only about 50 ms. Accordingly, each iteration takes only 60 ms, and three iterations sum up to 180 ms. Combined with the 210 ms of the first stage, the total processing time is 390 ms.

From the perspective of industrial dynamic scenarios and real-time feasibility, the overall processing time of 390 milliseconds can fully meet the physical interaction requirements of industrial dynamic adjustment or real-time correction for mid-to-low speed moving objects. Although it consumes an additional 175 milliseconds compared with the original open-loop method without optimization (215 milliseconds), in cases of severe object occlusion and cluttered backgrounds, this approach improves the grasping accuracy (ADD-S recall) from an unstable 79.6% to a reliable 91.7%. In practical factory sorting applications, blind pursuit of open-loop speed may result in object dropping or equipment damage due to unstable grasping, which would incur extremely high downtime for re-commissioning and hardware costs. Therefore, spending less than an extra 0.2 s to achieve more accurate and stable grasping is highly cost-effective, fully reasonable, and of great engineering practicality in real-world industrial dynamic grasping tasks.

#### 3.4.3. Visual Comparison of Results Across Different Methods

**Figure 8:**
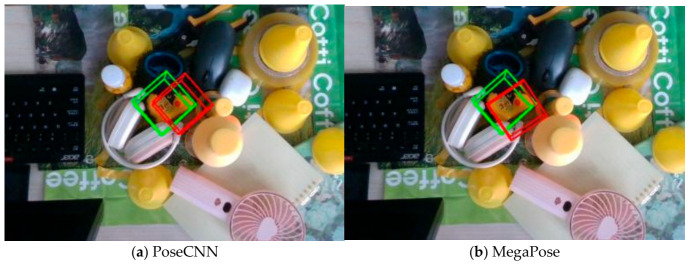

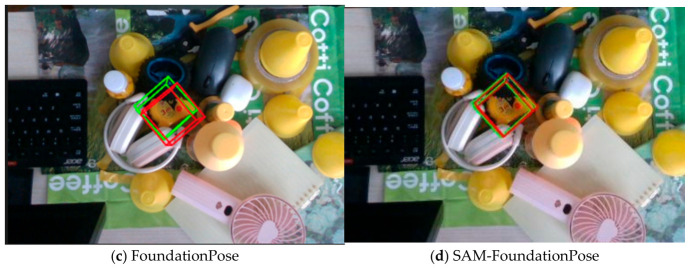
Comparison of 6D pose estimation of different algorithms. The green box represents the ground-truth pose of the object, and the red box represents the pose estimated by the proposed method.

To more intuitively analyze the sources of error for each method, Figure 7 presents a comparison of the 6D pose renderings of the above algorithms in a complex stacking scenario (with the tape measure as the target object). In the visualizations, the green 3D bounding box represents the ground-truth 6D pose of the target object, while the red 3D bounding box represents the actual estimated results of the different algorithms. As shown in Figure 8a, which presents the pose estimation result of PoseCNN under the complex stacking background, it can be observed that its red bounding box exhibits significant spatial offset and scale deviation from the green ground-truth bounding box. The position and orientation of the bounding box both fail to align with the actual physical boundaries of the target tape measure, reflecting that traditional methods are susceptible to the influence of surrounding interfering objects when the target is partially occluded, erroneously incorporating the occluded regions into the target feature extraction scope, which ultimately leads to pronounced drift in the pose estimation. Figure 8b presents the estimation result of MegaPose. Compared with PoseCNN, this method shows some improvement in the alignment of the bounding box; however, certain angular deviation and positional offset still remain. The bounding box fails to fully align with the edges of the target object, indicating that this method still has insufficient discriminative capability for the local features of the target in complex scenes and struggles to effectively resist background interference. Figure 8c presents the estimation result of FoundationPose. Its bounding box position is closer to the ground-truth pose compared to the previous two methods; however, a slight offset remains in the occluded edge regions of the target object. This indicates that, in the absence of precise target segmentation guidance, the method is still unable to fully overcome the feature confusion problem in complex scenes. As shown in Figure 8d, which presents the estimation result of the SAM-FoundationPose method proposed in this paper, the red bounding box nearly completely coincides with the green ground-truth bounding box. The bounding box accurately fits the physical edges of the target object, exhibiting high consistency with the ground truth in position, scale, and orientation. This fully validates that the closed-loop mask feedback mechanism can effectively isolate the true visible edges of the target object, effectively addressing the robustness bottleneck of unidirectional visual perception and demonstrating significantly improved pose estimation performance in complex scenes.

### 3.5. Core Module Ablation Experiments

To systematically verify the individual contributions and synergistic gains of each core module within the proposed SAM-FoundationPose framework on 6D pose estimation performance in cluttered stacking scenarios, this section conducts controlled-variable ablation experiments. The original FoundationPose is adopted as the baseline method, and multiple comparative variants are constructed by progressively removing key modules. Performance evaluations are carried out on the complex stacking background dataset to clarify the effectiveness of each module.

#### 3.5.1. Ablation Variant Design

To precisely quantify the marginal contribution of each module, the experiment follows the single-variable control principle and designs four progressive variants, with each variant modifying only one core configuration to ensure that any performance differences can be directly attributed to the target module. The design logic and specific configuration of each variant are as follows:

(1) Variant A (FoundationPose): Only the basic FoundationPose network is used without introducing any additional modules. This variant serves as the performance baseline, reflecting the inherent performance upper bound of the basic method under complex stacking backgrounds and providing a unified reference standard for all subsequent comparisons.
(2) Variant B (FoundationPose + SAM): Based on Variant A, the SAM segmentation module is introduced solely at the input end to provide an initial foreground mask, while maintaining an open-loop process without subsequent iterative optimization. This variant is used to verify the suppression effect of the SAM segmentation module on background interference.
(3) Variant C (FoundationPose + SAM + Iterative closed-loop): Based on Variant B, a closed-loop iterative mechanism is introduced; however, only a single “mask matching degree (IoU)” metric is used for confidence assessment, without adopting the multi-dimensional evaluation system proposed in this paper. This variant is used to verify the optimization capability of the closed-loop iterative mechanism and to compare the limitations imposed by single-metric evaluation on the iterative process.
(4) Variant D (FoundationPose + SAM + Iterative closed-loop + Multi-dimensional confidence assessment): The complete framework proposed in this paper, incorporating SAM segmentation, rendering-guided closed-loop iterative feedback, and multi-dimensional confidence assessment. This variant is used to verify the overall performance under the synergistic cooperation of all modules and to demonstrate the gain effect of multi-dimensional assessment on the closed-loop iteration.

#### 3.5.2. Ablation Experiment Results and Analysis

Based on the variant designs described above, we conducted performance tests on the ten categories of target objects on the complex stacking dataset. The comprehensive key indicators of each variant are presented in Table 4. The core evaluation metrics in the table include the ADD-S (<2 cm) recall rate and the average translation error. From the overall trend, as the SAM segmentation, closed-loop iteration, and multi-dimensional assessment modules are introduced sequentially, the model performance exhibits a marked stepwise improvement: the ADD-S metric gradually increases from the baseline of 72.4% to 91.7%, while the average translation error decreases from 16.8 mm to 3.5 mm, fully validating the positive contribution of each module to the pose estimation performance in complex scenes.

Regarding the SAM segmentation module, the experimental data show that after introducing the SAM segmentation prior, the ADD-S metric increases by 9.1%. This verifies that, under complex backgrounds, leveraging SAM’s high-quality segmentation capability to provide a clean region of interest (ROI) for pose estimation can effectively reduce the interference of background textures on geometric feature extraction, thereby enhancing the system’s robustness from the very source of perception.

Regarding mask feedback and closed-loop iteration, when comparing Variant B (only front-end SAM segmentation) with Variant C (basic closed-loop), the ADD-S recall increases from 81.5% to 86.3%, and the average translation error decreases from 11.2 mm to 6.5 mm. This significant performance gain verifies the core role of the rendered mask feedback. In complex stacked scenes, a single run of SAM (Variant B) often produces over-segmentation due to low edge contrast, i.e., mistakenly classifying background clutter as the target object. With the introduction of 3D rendering priors, the mask feedback provides explicit geometric constraints, eliminating background pixels inconsistent with the target shape, thereby achieving more accurate pose convergence.

For the confidence metric, we compare Variant C (single metric using only IoU mask matching) with Variant D (multi-dimensional confidence system). Tests show that when the system relies solely on the single 2D mask matching metric (IoU) for evaluation (Variant C), its ADD-S is only 86.3%. This is because the pure 2D IoU metric suffers from a “depth blind spot” and “pose ambiguity” for symmetric objects. When the pose drifts forward or backward along the camera optical axis (*Z*-axis), the rendered mask on the 2D plane may still highly overlap with the real object, causing the single confidence metric to terminate the adjustment prematurely. The multi-dimensional confidence proposed in this framework (integrating reprojection error, geometric depth error, and mask matching) effectively solves this problem. Through cross-verification of 3D spatial information, the system continues to adjust until the object boundaries are fully aligned, ultimately raising the recall rate to 91.7%.

To further analyze the evolution of the closed-loop system during computation, we statistically investigated the variations in the overall ADD-S recall rate and computational latency with an increasing number of iterations k, based on the cluttered and stacked dataset. Experimental results show that at k = 0 (the zero-order initial perception stage), although SAM performs preliminary background filtering, the ADD-S recall is only 81.5% due to severe dense occlusion. When the system executes the first round of rendered mask feedback (k = 1), the introduced geometric constraints free the system from interference caused by erroneous estimations, and the accuracy rapidly rises to 84.5%. In the subsequent 2nd and 3rd iterations, the system mainly conducts millimeter-level fine alignment in the *Z*-axis depth and minor rotation angles, with the recall rate gradually increasing to 88.2% and 90.9%, respectively. When the number of iterations reaches k = 4, the ADD-S recall reaches 91.7% and tends to stabilize. Thereafter, even if the number of iterations continues to increase (k ≥ 5), the accuracy barely improves (by less than 0.1%), while the computational latency increases significantly. This demonstrates that the proposed closed-loop architecture achieves high computational efficiency. In practical applications, only 3 to 4 iterations (3 on average) are generally required to achieve a favorable balance between operational accuracy and execution speed.

In addition, we analyzed the influence of the comprehensive confidence exit threshold τ on system performance and computational cost. To verify the rationality of the threshold setting in this paper, we conducted comparative tests on unstructured stacked datasets with the same difficulty, using a lenient threshold (τ = 75), a standard threshold (τ = 85), the default in this paper), and a strict threshold (τ = 95). The test results show that: For the lenient threshold (τ = 75), the system easily satisfies the exit condition and terminates after only 1.8 iterations on average. Although the computation time is extremely short, the system often stops adjusting prematurely before completely eliminating edge position deviations, resulting in a final ADD-S recall of only 83.2%, which fails to meet the requirements of high-precision grasping. For the standard threshold (τ = 85), the system achieves the best balance, with the average number of iterations stabilizing at around 4. While effectively avoiding large positional deviations, it firmly maintains the ADD-S recall at a high level of 91.7%. For the strict threshold (τ = 95), due to the inherent hardware noise of depth cameras and edge degradation of certain materials, it is difficult for the system to achieve perfect consistency. This leads to excessive iterative computation, and the system is often forced to stop only when reaching the preset maximum number of iterations ($k_{max}=10$). This introduces significant computational latency, yet the final ADD-S recall only slightly increases to 91.9%, yielding very little gain at the cost of much extra time. The above tests demonstrate that the selection of τ = 85 is fully supported by experimental data, and also validate that the multi-dimensional metric can effectively regulate the iterative process.

#### 3.5.3. Ablation Experiment Conclusion

The ablation experiment results fully demonstrate that the SAM prior, the closed-loop iterative mechanism, and the multi-dimensional confidence assessment module designed in this paper play complementary and irreplaceable roles in improving 6D pose estimation accuracy. Their synergistic effect enables the proposed framework to achieve a stepwise leap in 6D pose estimation performance under complex stacking scenarios, which not only validates the effectiveness of each module but also demonstrates the rationality and robustness of the overall framework design, providing reliable technical support for subsequent practical applications such as robotic grasping.

### 3.6. Verification of Robotic Arm Visual Grasping in Real-World Scenarios

To further verify the reliability of the proposed SAM-FoundationPose-based iterative closed-loop fusion framework in actual physical interactions, this section conducts an autonomous robotic grasping experiment in a real unstructured environment.

#### 3.6.1. Grasping Experiment Procedure

**Figure 9:**
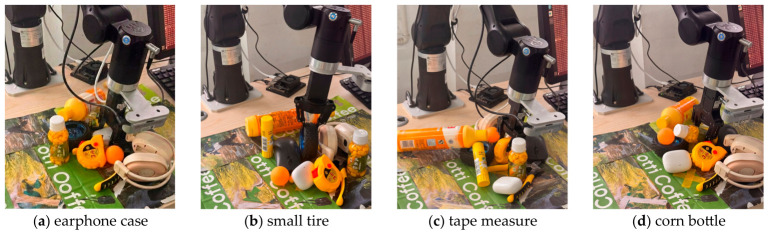
Grasping results of the robotic arm for different targets.

The execution logic of the system is as follows: First, the camera captures the global observation data from the current viewpoint, and the SAM-FPose framework performs initial pose estimation. If the multi-dimensional confidence score does not meet the threshold, the system activates the mask rendering feedback mechanism for iterative correction. After the pose estimation is completed, the hand-eye transformation matrix is used to map the target pose into the robotic arm’s coordinate system, driving the end effector to perform trajectory planning and closing actions. Figure 9 intuitively demonstrates the physical results of the system successfully guiding the robotic arm to grasp multiple target objects in a highly interfered environment. The figure respectively shows the grasping states for objects with different geometric features and surface textures, including (a) earphone case, (b) small tire, (c) tape measure, and (d) corn bottle. It can be observed that even when the target objects are located in severely occluded and unstructured cluttered areas with complex backgrounds, the proposed framework can still output pose estimation results that closely match the ground truth, providing reliable guidance for the robotic arm to generate collision-free motion trajectories, approach precisely, and robustly grasp the targets. This fully verifies that the closed-loop iterative correction mechanism can effectively rectify single-shot perception deviations and significantly enhance the stability and reliability of actual physical interactions.

#### 3.6.2. Grasping Result Analysis

**Figure 10:**
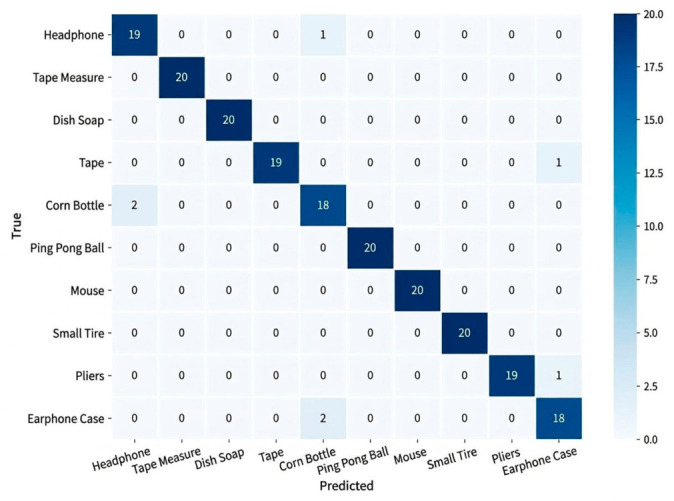
Statistics of robotic arm grasping results.

The experiment conducted 20 independent grasping trials for each of the 10 categories of target objects (with the scene re-scrambled for each trial to ensure random configurations), cumulatively performing 200 attempts. The detailed quantitative statistical results are shown in Figure 10. The experimental data indicate that the system successfully completed 193 out of the 200 grasping tasks, achieving an overall grasping success rate of 96.5%. As can be seen from the confusion matrix in Figure 10, the majority of objects achieved a grasping success rate of 100%. For easily confusable objects such as adhesive tape, whose edges are susceptible to interference from surrounding items and background, the system exhibited a few grasping failure cases; however, the grasping success rate remained above 90%. To a certain extent, this result indicates that the iterative feedback mechanism can effectively improve the system’s physical execution robustness in interfered environments, providing reliable technical support for robotic grasping tasks in complex scenes.

To comprehensively investigate the robustness of the proposed framework in extremely unstructured environments, this section analyzes all seven failures that occurred during 200 real-world physical grasping validations. The results show that the system failures are strictly concentrated in the following specific rigid-body categories: corn bottle (2 times), earphone case (2 times), over-ear headphone (1 time), insulating tape (1 time), and pliers (1 time). The main causes of failure are analyzed as follows: (1) Under severe interference from multi-source physical lighting, specular highlights appear on the rigid-body surface. These strong visual noises completely erase the original texture and color gradient of the object, causing the front-end large model SAM to suffer severe oversaturation and blunting when receiving pose-rendered guidance prompts, resulting in large irreversible topological holes inside the binary mask. During region-of-interest (ROI) cropping with such incomplete masks, the downstream FoundationPose inference engine loses more than 40% of the effective surface features of the rigid body. Due to the severe skewness of the feature space, the finally output 6D pose drifts significantly along the camera optical axis, leading to empty grasping by the end-effector. (2) The color contrast between the outer contour of the rigid body and the ambient desktop background is extremely low, causing severe degradation of the object’s physical edges in the image space. In this case, even with closed-loop feedback guidance, SAM produces semantic ambiguity in edge-conflict regions, causing the mask boundary to “leak” outward and incorrectly incorporate surrounding non-target cluttered background pixels into the target region, forming severe regional adhesion and artifacts. Such “adhesive masks” containing high-entropy noise break the original rigid-body geometric topological constraints of the object, introducing background features into pose estimation during feature matching. This ultimately leads to deflection in the output pose, causing the rigid body to slip due to uneven force at the moment the robotic arm closes and locks.

## 4. Discussion

### 4.1. Innovations of This Work

Traditional 6D pose estimation algorithms predominantly adopt an open-loop control strategy of “segmentation first, then estimation.” This approach is highly dependent on the quality of front-end segmentation; once segmentation fails due to complex backgrounds or severe occlusions, errors are irreversibly propagated downstream and amplified. Classic rendering-based pose optimization methods (e.g., ICP algorithms) often rely on registration driven by local geometric gradients and are highly prone to falling into local optima in textureless regions or under severe occlusions. The proposed framework is fundamentally different from existing methods such as iterative pose-refinement, render-and-compare, and analysis-by-synthesis. Existing classical iterative fine-tuning or analysis-by-synthesis methods typically perform unidirectional approximation within the pose space or image feature space. They render the 3D model of the object at the current predicted pose, and then update the 3D pose stepwise by directly computing or network-predicting the pose residual based on the feature discrepancy between the rendered view and the real observation. A common limitation of such methods is that their optimization process cannot back-propagate to refine the input 2D image segmentation mask. In unstructured environments with dense stacking, severe occlusion, or high color similarity between the target and the background, if the initial 2D mask from the front end contains surrounding clutter or background pixels due to blurred boundaries, traditional render-and-compare methods will only force geometric alignment within this erroneous region contaminated by heavy noise. This makes them prone to local optima and unable to achieve accurate error elimination.

In contrast, the algorithmic innovation of the closed-loop framework proposed in this paper lies in the construction of a cross-dimensional alternating correction mechanism. The system converts the currently predicted imperfect 3D pose into a 2D geometric region mask. Its core purpose is not to directly superimpose an update step on the 3D pose, but to feed this region mask as a definite spatial geometric constraint back into the prompt encoder of the 2D vision foundation model (SAM). This mechanism leverages the rigid-body geometric invariance of the object to force the perception front-end to re-optimize the target boundary in disturbed edge-conflict regions. Through such an alternating closed loop, the system dynamically cleanses and purifies the feature input source while optimizing the pose. When the pose estimation engine obtains clean local features completely free of background noise in the next iteration, it can directly eliminate the matching drift commonly encountered in traditional methods. This closed-loop control scheme—in which geometric priors cleanse semantic inputs, and purified semantics guide geometric estimation—is fundamentally different in algorithm logic from conventional engineering combinations and single-space pose fine-tuning methods.

In addition, it is necessary to discuss the technical route adopted in this paper within the context of modern robotic manipulation. At present, tasks such as cluttered scene cleaning and random bin picking are often formulated as end-to-end Deep Reinforcement Learning (DRL) problems. In the study proposed by Turco et al. [37] robots can learn optimal grasping and obstacle-clearing strategies through direct interaction with the environment, and can leverage well-designed hardware grippers to reduce algorithmic complexity. Although reinforcement learning methods exhibit excellent adaptive capabilities, this research still holds significant engineering value, mainly for the following two reasons: (1) Data Efficiency and Virtual-to-Real Transfer Issues: End-to-end policies typically require extensive trial-and-error interaction data and high-precision physics simulation environments. When deploying well-trained policies from simulation to real robots, they are highly susceptible to the gap of sim-to-real, which leads to significant degradation in grasping performance. In contrast, the proposed framework directly relies on the zero-shot generalization ability of visual models. Given the 3D model of a known object, it can be employed in a plug-and-play manner without any pre-training or fine-tuning for specific environments during real-world physical deployment. (2) Industrial Physical Safety and Interpretability: In precision sorting of high-value, fragile, or heavy-duty workpieces, reinforcement learning, as a black-box network, poses risks of physical collisions and workpiece damage if unknown actions generated during the exploration phase are directly applied in real production. In contrast, the deterministic geometric feedback proposed in this paper incorporates multi-dimensional confidence evaluation and a strict rollback mechanism, which together act as a gate for control actions, ensuring safe, controllable, and highly predictable physical behaviors of the robot.

### 4.2. Limitations of This Work

Although the proposed system demonstrates high perceptual accuracy and 3D spatial alignment capability in unstructured scenes, there remain non-negligible engineering limitations. The pose estimation of the FoundationPose module strictly relies on the 3D model of the target object as an input prior. When confronted with open-world targets for which a 3D model is completely unknown or unavailable, the applicability of the framework will be constrained. To overcome this limitation in real-world open scenarios lacking 3D data or in complex random bin picking tasks, two promising technical routes can be explored in the subsequent iterative research of this framework: (1) The system can integrate advanced zero-shot 3D reconstruction foundation models to directly infer explicit meshes or neural implicit surface geometries of unknown objects online using real-time multi-view observations at the work site, thereby dynamically generating alternative geometric priors. (2) The framework can introduce category-level shape priors, enabling the system to adaptively deform and map a general standard shape template to novel instances within the same semantic category under the constraint of known semantic classes, thus completely eliminating the absolute dependence on instance-specific 3D models.

Besides the aforementioned dependence on specific 3D models, this method also has the following clear limitations in real-world engineering deployment: (1) Computational overhead and high-frequency real-time constraints: This framework achieves high precision through multiple “rendering–feedback–re-segmentation” loops, with the system taking an average of 390 ms to process one object. Although this speed fully meets physical interaction requirements for medium–low-speed industrial sorting and quasi-static operations, the indispensable computational overhead of SAM and FoundationPose in each iteration (approximately 60 ms per fine-tuning loop) prevents the system from being directly applied to ultra-high-speed dynamic tracking or flying grasping tasks requiring frame rates above 30 FPS. (2) Physical robustness boundary under extreme occlusion: The closed-loop iterative mechanism can effectively correct pose drift caused by moderate occlusion, yet the system still has an insurmountable robustness limit. When the target object is heavily occluded by surrounding clutter such that its physical surface occlusion exceeds 60%, the actually captured RGB features and depth point clouds become extremely sparse. In such extreme cases, due to the insufficient information available for feature matching, different erroneous poses may produce highly similar incomplete masks after projection, causing misjudgment in the multi-dimensional confidence evaluation module and making the algorithm prone to converging to a physically incorrect local optimum.

## 5. Conclusions

Addressing the degradation in 6D pose estimation accuracy and insufficient robustness caused by object occlusions and background texture interference in complex unstructured scenes, this paper proposes an iterative closed-loop pose estimation framework based on the deep coupling of SAM and FoundationPose. The framework achieves accurate extraction of target regions by introducing the SAM zero-shot segmentation prior, constructs a closed-loop iterative correction mechanism based on mask rendering feedback, and designs a multi-dimensional confidence assessment system as the iteration termination criterion, systematically resolving the visual perception degradation problems under complex stacking, physical occlusions, and cluttered texture interference. In multiple sets of experimental validations in real-world scenes, the proposed method improves the 6D pose estimation accuracy while maintaining the zero-shot generalization advantage of FoundationPose, with the ADD-S metric improved by 19.3% over the baseline and the average translation error reduced to 3.5 mm. In real robotic arm grasping verification, the system achieves an overall grasping success rate of 96.5% in unstructured scenes with strong interference, validating the full-pipeline reliability from perception estimation to physical interaction. This research requires no large-scale annotated data, effectively overcomes the robustness bottleneck of traditional open-loop methods with single-shot perception, and provides a visual perception solution that combines zero-shot generalization capability with high accuracy and robustness for tasks such as industrial random bin picking and household object manipulation, while also offering a referable closed-loop optimization approach for model-prior-based pose estimation optimization.

## Figures and Tables

**Table 1:** SAM-FoundationPose Iterative Closed-Loop Optimization.

Input: RGB-D image I, 3D model M_cad_, camera intrinsics K
Output: Optimal 6D pose T*
Parameters: τ ← 85 (confidence threshold), k_max_ ← 10 (maximum iterations)
1: k ← 0
2://Initialization phase
3: bbox ← YOLOv5n(I) ▷ Heuristic bounding box prompt
4: M_0_ ← SAM(I, bbox) ▷ Initial foreground mask
5: T_0_ ← FoundationPose(I, M_0_, M_3d_) ▷ Initial 6D pose estimation
6: S_0_ ← ComputeScore(T_0_, M_0_, I, M_3d_, K) ▷ Multi-dimensional confidence score
7://Iterative refinement phase
8: while S_k_ < τ and k < k_max_ do
9: k ← k + 1
10: M_guide_ ← RenderMask(T_k_ − 1, M_3d_, K) ▷ Render geometric prior mask
11: M_k_ ← SAM(I, M_guide_) ▷ Mask refinement with spatial prior
12: T_k_ ← FoundationPose(I, M_k_, M_3d_) ▷ Pose re-estimation with refined mask
13: S_k_ ← ComputeScore(T_k_, M_k_, I, M_3d_, K) ▷ Update confidence score
14: end while
15: return T* ← T_k_

**Table 2:** Quantitative results of pose estimation in cluttered scenes.

Target Object (Category)	Object Type Characteristics	ADD(-S) Recall Rate (%)	Average Translation Error (mm)	Average Rotation Error (°)
Headphones	Heterogeneous shape/background texture	98.2	3.2 ± 0.6	1.9 ± 0.4
Tape measure	Same-color background confusion	97.5	4.1 ± 0.9	2.3 ± 0.5
Detergent bottle	Cylindrical symmetry/high-frequency reflections	96.8	3.8 ± 0.7	2.1 ± 0.4
Adhesive tape	Low-contrast edges	94.1	5.2 ± 1.2	3.4 ± 0.8
Corn bottle	Complex internal texture	98.0	2.9 ± 0.5	1.7 ± 0.3
Ping-pong ball	Textureless solid-color sphere	99.2	1.8 ± 0.3	1.2 ± 0.2
Computer mouse	Weak texture/dark color	98.5	2.6 ± 0.4	1.8 ± 0.4
Small tire	Strong geometric jagged texture	97.4	3.5 ± 0.6	2.0 ± 0.4
Pliers	Slender irregular structure	96.1	4.4 ± 1.0	2.6 ± 0.6
Earphone case	Multi-source lighting/specular reflections	95.3	4.8 ± 1.1	3.1 ± 0.7
Mean	-	97.1	3.63 ± 0.73	2.21 ± 0.47

**Table 3:** Comparative results of different algorithms under cluttered stacking backgrounds.

Method	Input Data Type	ADD-S Recall Rate (%)	Average Translation Error(mm)	Average Rotation Error (°)	Average Inference Time(ms)
PoseCNN	RGB	45.2	35.6 ± 8.4	22.4 ± 5.1	45
MegaPose	RGB-D	71.4	19.5 ± 4.2	11.2 ± 2.8	385
FoundationPose	RGB-D	79.6	14.2 ± 3.1	8.7 ± 1.9	215
Ours (SAM-FoundationPose)	RGB-D	91.7	3.5 ± 0.7	2.1 ± 0.4	390

**Table 4:** Comparative results of core module ablation experiments.

Experimental Variant	SAM Segmentation	Closed-Loop Iteration	Multi-Dimensional Assessment	ADD-S (%)	Average Translation Error (mm)
A	×	×	×	72.4	16.8
B	√	×	×	81.5	11.2
C	√	√	×	86.3	6.5
D (Ours)	√	√	√	91.7	3.5

## Data Availability

Data are contained within the article.
